# Married men’s sexual and reproductive health concerns and related health-seeking behavior in Bangladesh: A mixed methods study

**DOI:** 10.1186/s41043-022-00313-x

**Published:** 2022-08-30

**Authors:** Raia Azmi, Ilias Mahmud, Kuhel Faizul Islam, Md Tanvir Hasan, Sabina Faiz Rashid

**Affiliations:** 1 Center for Gender, Sexual and Reproductive Health and Rights, BRAC James P Grant School of Public Health, BRAC University, 5th Floor (Level-6), icddr,b Building, 68 Shahid Tajuddin Ahmed Sharani, Mohakhali, Dhaka, 1212 Bangladesh; 2 Department of Public Health, College of Public Health and Health Informatics, Qassim University, Al Bukairiyah, Saudi Arabia

**Keywords:** Sexual and reproductive health, Married men, Sexually transmitted illnesses, Bangladesh

## Abstract

**Background:**

In Bangladesh, men’s sexual and reproductive health (SRH) needs and related services are often neglected. Little is known of men’s SRH concerns, and of the phenomenal growth of the informal and private health actors in the provision of sexual health services to men in rural and urban areas of Bangladesh.

**Methods:**

Using a mixed methods approach, a survey of 311 married men in three rural and urban sites was conducted in three different districts of Bangladesh and 60 in-depth interviews were conducted to understand their SRH concerns and choice of providers to seek treatment.

**Results:**

The research findings reveal that- men’s various SRH concerns are embedded in psychosocial and cultural concerns about their masculinity and expectations of themselves as sexual beings, with worries about performance, loss of semen and virility being dominant concerns. Sexually transmitted infections (STIs) were also mentioned as a concern but ranked much lower. Informal providers such as village doctors (rural medical practitioners and *palli chikitsoks*), drug store salespeople, homeopaths, traditional healers (*Ojha/pir/fakir, kabiraj, totka*) and street sellers of medicines are popular, accessible and dominate the supply chain.

**Conclusion:**

There is a need of appropriate interventions to address men’s anxieties and worries about their sexual abilities, well-being and choice of providers. This would go a long way to address and alleviate concerns, as well as identify and push men to seek  formal care for asymptomatic STIs, and thereby reduce costs incurred and gender tensions in households.

## Introduction

Sexual health problems encompass a wide range of conditions including sexually transmitted infections/diseases (STIs/STDs) and syndromes impairing sexual function and conditions related to reproduction [1]. Sexual and reproductive ill-health is associated with morbidity, not just mortality, and has a trigger effect on children and families, and these are harder to quantify [2, 3]. Thus, there is a need for health research and practice that is gender sensitive in relation to men’s lives and to understand masculinities in relation to reproductive health and illness [4]. However, the sexual and reproductive health (SRH) of men in low-income countries has received very little attention from the research community, and public sector health care planners and providers [5].

Men’s involvement has been described as ‘critical for effectiveness of SRH programs,’ because men are often the ‘gatekeepers’ of women’s economic and social mobility, including their care seeking [6]. The International Conference on Population and Development (ICPD) emphasized the participation of both men and women in SRH programs to achieve high standards of sexual and reproductive health and rights (SRHR) for all [6, 7]. South Asian society contexts, characterized by high levels of poverty, pervasive systems of patriarchy and heteronormativity, emphasize the need for understanding and involving men in the discourse of SRHR and prevention of STIs [8]. A growing body of research reports specific sexual health concerns of men in the South Asian contexts. These included concerns over ejaculation, penis size, erectile dysfunction, STIs, impotence, nocturnal emissions, short duration of intercourse and similar psychosexual issues [9–13].

Various factors were found to be associated with men not seeking health care for SRH conditions in research conducted globally. These include- lack of knowledge and awareness, stigmatization and negative attitudes that persist in society against STDs/STIs, limited financial resources, reluctance to disclose SRH concerns to sexual partners/wife, and lack of good quality services [14–16]. When care is sought, it is usually sought first from informal allopathic practitioners or traditional healers before moving on to formal healthcare services in private and public facilities [15, 17, 18]. While majority of the research and discussion on SRHR, especially in South Asian literature, reports men as the ‘problem,’ ‘risky’ or ‘background figure,’ there are some literature that aim to understand men’s behavior as a means to improve SRH. Hegemonic masculinity was reported as an underlying factor that influence sexual behavior as well as health seeking in men in the studies, where the men do not seek care readily because it is seen as a sign of weakness, would not disclose STIs to female partners because they want to keep assuming the dominant role in relationships, and control contraceptive use [8, 9, 12, 19]. It was also reported that men who routinely perpetrated violence against women (VAW) in marital relationships were even less likely to have disclosed STI conditions to their partners, compared to other men not committing VAW [20].

To attain universal health coverage (UHC), a key health target of the Sustainable Development Goals (SDGs) identified in the 2030 Agenda [21], ensuring access to appropriate SRH services and promotion of SRH services to both men and women are essential [21]. The ‘Global health sector strategy on STIs, 2016 -2021,’ adopted by the World Health Assembly in 2016, situates control and prevention of STIs as critical to the achievement of UHC [21]. The strategy urges a people-centered approach based in principles of human rights, gender equality and health equity, and calls for a comprehensive, evidence-based combined behavioral, biomedical and structural prevention efforts to improve SRH of population. Therefore, it is very important to focus on social and cultural understandings of SRH and related concerns affecting men and consider in-depth understanding of men’s behaviors and practices related to SRH and relevant care seeking to design effective SRH interventions and STIs prevention strategies.

Since there is very little research evidence available on SRH of men in Bangladesh, this study was undertaken- to explore the SRH concerns of men, their perspectives on SRH-related conditions, how hegemonic masculinity shapes these concerns and related health-seeking behavior- in order to add value to the evidence-base, so that government and non-government organizations may uptake the study findings and utilize them to inform health strategies and design interventions to improve SRH of men, and thereby women as well, in the long run.

## Methods

This paper is out of a larger explanatory sequential mixed methods (quan → QUAL) study involving a cross-sectional survey and qualitative in-depth interviews (IDIs) of married men, women and SRH service providers (both formal and informal) we conducted to understand married men’s and women’s SRH concerns, their SRH sufferings and provision of SRH services in three areas of Bangladesh—Gangachara sub-district of Rangpur division, Jagannathpur sub-district of Sylhet division and Motijharna slum of Chattagram division. The larger study recruited 312 ever-married women aged 15–49 years, 311 ever-married men aged 15–49 years and 303 health care providers for the survey [22]. This paper uses data of 311 ever-married men who had participated in the survey and 60 ever-married men who had participated in qualitative in-depth interviews. The main purpose of the quantitative survey was to estimate the prevalence of self-reported sexual and reproductive health conditions and concerns suffered by married men in the study sites and examine their health-seeking patterns and formal and informal health care service utilization. The objectives of the qualitative interviews were to elaborate the quantitative findings and further explore where men go for SRH related problems, how hegemonic masculinity shapes their SRH concerns and how their perceptions on sexual health influence their family life. The combination of qualitative and quantitative approaches (survey and in-depth interviews) strengthens the study and its conclusion.

The quantitative survey was conducted during July 2008 and January 2009. We interviewed at least 100 men from each of the three study sites to ensure good representation of ever-married men from a diverse socioeconomic background and geographical areas (three divisions) of Bangladesh. Before starting of the quantitative survey, complete household listings of the three study sites were obtained from the Union Parishad offices (local Govt. administrative office). A systematic sampling procedure was then used to recruit participants for the survey. In Gangachara and Jagannathpur sub-districts, participants were recruited from every fifth households, whereas in Motijharna slum participants were recruited from every tenth household due to high household density. Participants from Gangachara sub-district represent the ultra-poor from the northern part of Bangladesh repeatedly affected by seasonal food insecurity [23]. Participants from Jagannathpur sub-district represent rural poor from the south-east part of Bangladesh; this region is a religiously conservative and high fertility area [24]. Those from Motijharna slum represent typical urban slum population of Bangladesh. These sites were chosen based on the fact that limited research has been conducted on SRH issues of married men living in the disadvantaged and rural communities in Bangladesh. Participants for the qualitative phase were recruited through purposive sampling and they were chosen based on the response in the survey. Self-reported SRH sufferings, perceived causes and severity of the SRH problems and health-seeking behavior for the reported SRH concerns and public and private health care service utilization were considered as the selection criteria for recruiting the in-depth interview participants.

Data were collected on a range of issues such as married men’s knowledge about SRH, their SRH sufferings in the last 12 months, perceived causes and severity of the SRH problems, public and private health service utilization, cost associated with treatment and the providers used. Five male data collectors supervised by a researcher of BRAC James P Grant School of Public Health (BRAC JPGSPH), BRAC University, facilitated the data collection process. Face-to-face interviews were conducted in a safe place convenient for the participants, and no financial or material incentives were provided. Informed consent was obtained from all the participants. The researchers maintained strict privacy and confidentiality by de-identifying the data and protecting data in a locked cabinet. Only the researchers and data analysts had access to the data. The study received ethical clearance from the ethical review committee of BRAC JPGSPH, BRAC University.

Quantitative data were analyzed using Statistical Package for the Social Sciences (SPSS) software. Appropriate descriptive statistics (frequency and percentage for categorical variables and mean and standard deviation for continuous variables) were obtained after examining outliers and skewness of the different variables. Qualitative data were coded using Atlas ti; coding was both inductive and deductive. To analyze, data display matrices were generated, thematic segregation of data in the matrices and thematic analysis was done. Identified patterns, unusual responses and reportable findings were drawn out. Researchers were reflexive throughout data collection and analysis, and tried to maintain objective views and keep aside own judgments and bias during analysis. Inter (83%)- and intra-coder (85%) reliability checks were done to ensure validity of findings.

## Results

### Characteristics of the study participants

Table 1 shows characteristics of the survey participants. The mean age of the participants was 35.6 years with a standard deviation of 8.2 years. Most of them (36.7%) were from the age group 31–40 years followed by 34.1% from the age group 21–30 years. All of them were (ever) married and sexually active at the time of the survey. Over a quarter (26%) of the participants got married by the age of 20 years and by the age of 25 years, over half (51.1%) of them got married. More than a third of the participants (34.1%) did not have any formal education, while only about 10% had completed secondary or higher education. At the time of the survey, only 2.3% participants were unemployed, while little less than a third (32.2%) were working as an unskilled or semiskilled labor and a quarter (24.1%) were running small business.

**Table 1:** Socio-demographic characteristics of the survey participants (*n* = 311) and qualitative in-depth interviews participants (*n* = 60)

Variables	Survey participants (*n* = 311)		Qualitative in-depth interviews participants (*n* = 60)
	Frequency (%)	Mean (SD)	Frequency
Age		35.59 (8.16)	
18–20 Years	3 (1)		1
21–30 Years	106 (34.1)		24
31–40 Years	114 (36.7)		24
41–50 Years	88 (28.3)		11
Age at first marriage^a^		23.61 (4.30)	
By age 17 year	18 (5.8)		
By age 20	81 (26.0)		
By the age 25	159 (51.1)		
By the age 30	292 (93.9)		
By the age 42	311 (100.0)		
Education		4.14 (4.03)	
No education	106 (34.1)		15
Primary incomplete	61 (19.6)		16
Primary complete	39 (12.5)		7
Secondary incomplete	75 (24.1)		18
Secondary complete or higher	30 (9.6)		4
Area of residence			
Gangachara Upazila	107 (34.4)		18
Jagannathpur Upazila	104 (33.4)		23
Motijharna slum	100 (32.3)		19

SD indicates standard deviation

^a^The age at first marriage is defined as the age at which the respondent married his first wife.

Among the qualitative participants, most (20 out of 60) were from the age group 25–29 years followed by 15 from the age group 20–24 years. Out of 60 qualitative participants, 18 were from Gangachara Upazila, 23 from Jagannathpur Upazila and 19 from Motijharna slum (Table 1).

### Participant’s self-reported SRH concerns and illnesses

During survey and qualitative in-depth interviews, we asked the participants to report common SRH concerns and illnesses they were suffering from in the last 12 months. Of the 311 participants interviewed in the survey, 48.6% (151/311) reported having at least one SRH concern/illness in the preceding 12 months. Among those reported suffering from any concern/illness, almost a quarter (24.5%) reported suffering from frequent urination/incontinence followed by 23.2% reported suffering from burning or pain when urinating. About 13% of them reported suffering from itching or burning in the genital area and 1.3% reported suffering from bumps or sores in the genital area (Table 2). The qualitative findings suggest that participants commonly suffered from: itching in the genital area and groins due to scabies (parasitic skin infection); *daud* or ringworm infection on the skin; itchy skin rashes due to eczema; penile discharge of pus/blood, and, sores and ulcers in the genital area and penis due to STDs; and burning sensation during passing of urine, frequent passing of urine and pain in lower belly due to urinary tract infections (UTIs).

**Table 2:** Self-reported SRH concerns of the study participants (*n* = 151)

Individual SRH concerns*	Frequency (percentage)	Percent of cases (*n* = 151)
Shortened duration of sexual intercourse	51 (18.1)	33.8%
Frequent urination/incontinence	37 (13.1)	24.5%
Loss of semen before and after urination	36 (12.8)	23.8%
Burning or pain when urinating	35 (12.4)	23.2%
Nocturnal emissions	23 (8.2)	15.2%
Impotence/difficulty to maintain an erection	22 (7.8)	14.6%
Itching or burning in genital areas	19 (6.7)	12.6%
(Ekshira)	13 (4.6)	8.6%
Ejaculation before coitus	12 (4.3)	7.9%
Anxieties about penis size	6 (2.1)	4.0%
Discharge from penis	4 (1.4)	2.6%
Worries about masturbation	2 (0.7)	1.3%
Pain during sexual intercourse	2 (0.7)	1.3%
Loss of semen/dhatu	2 (0.7)	1.3%
Bumps or sores in genital areas	2 (0.7)	1.3%
Pain in the testicles	1 (0.4)	.7%
Others	15 (5.3)	9.9%

*Multiple responses

In the survey, one-third (33.8%) of the 151 participants, who had a concern, reported that inability to have sexual intercourse for long is a major concern for them followed by a quarter (23.8%) who reported loss of semen before and after urination as a concern (Table 2). The qualitative findings revealed the following psychosexual concerns: shortened duration of intercourse, loss of semen before and after urination, thin/poor amount of discharged semen, premature ejaculation, inability to get an erection, lack of excitement during intercourse and nocturnal emissions. Therefore, both qualitative and quantitative findings suggest that many of the participants suffer from psychosexual anxieties, which are viewed as serious concerns and a source of perpetual anxiety and stress.

### Perceived causes of the concerns

Qualitative findings suggest that many of the participants had various understandings about the causes of their SRH illnesses, which did not correlate with medical causes. One participant, 27-year-old a *halua* (sweets) maker, said:

I have to stand close to a stove all day to make halua (a dessert popular in Bangladesh). The heat burns my lower belly. I think this is why it burns so much during urination. It gets a lot worse during summer, when the heat of the room adds to the heat of the stove.

The above quote suggests that the participant had misconception that exposure to excessive heat or warm conditions, such as summer heat, causes and exacerbates UTIs, genital sores and related itching.

Qualitative data also revealed that masturbation was thought to be an illness itself by majority of the participants, and habit of masturbating during young age was thought to be the cause of many SRH illnesses that manifest in future, which include shortened duration of intercourse, loss of semen before and after urination, thin/poor amount of discharged semen, premature ejaculation, inability to get an erection, lack of excitement during intercourse and nocturnal emissions. One participant, 25-year-old rickshaw puller, reported:

I used to masturbate a lot when I was young. I could not control my urges to have sex and would masturbate frequently. It was 10-12 years ago. After marriage, I realized I could not hold it in for long during sex. I tried a lot, but it seems my bad habit has drained me of my strength.

The above case shows how this person is assuming that masturbation is causing the shortened duration of intercourse. His case study is typical of many cases we found in interviews of poor men, who believe that their habit of masturbation in young life leads to many sexual illnesses.

In the qualitative interviews, most of the men appear to be well informed about the causes related to STDs/STIs. Almost all of them mentioned that sex with multiple partners is a reason of STD/STI. A 28-year-old man who had open sores in his genitals said:

I went to bad women (prostitutes) to have sex that’s why I’ve got this problem [discharge of pus from penis].

Extramarital sex was also perceived as the cause of other problems. One participant, 28-year-old carpenter, reported:

I have had sex with a woman other than my wife even after my marriage that’s why I have got this problem of premature ejaculation.

The above quotes highlight how some sexual concerns are linked to shame and guilt about extramarital affairs, and going to prostitutes.

### Perception of and attitude toward SRH illnesses: how ideals of masculinity shape them

The qualitative findings revealed that SRH sufferings and illnesses were considered as shameful by the study participants. Sex was considered a ‘taboo’ subject, and conditions/illnesses related to sex life/sexual organs were deemed disgraceful (*lojjar bishoy*) if exposed. One participant, 25-year-old three-wheeler vehicle driver, said:

These illnesses are secret illnesses because you don’t want anyone to know you have it; it is very shameful. All the men want to keep it hidden. Even I did so. Only my wife knew because I could not keep it from her.

The above case shows how the participant kept his illnesses secret because he thought these illnesses are shameful and should not be shared with others. This case study is typical of many case studies, and because of this social stigma, many reported delaying seeking care, especially formal care as long as the conditions are manageable.

Misconception such as frequently visiting prostitutes was seen to cause sexual illnesses, which can reduce sexual prowess and pleasure, leading to stigmatization and being viewed as a ‘bad person’ (*kharap chele*). This also implied that the illnesses were the individual’s fault for having a bad character. A participant, 42-year-old shop-owner, said:

My nephew is newly married. He is having trouble of shortened duration of sex with his wife. He has also become very weak. We are very confused because these are all signs of a ‘kharap chele’. But he was always a good boy who never visited prostitutes. We are bewildered at what to do.

The above quote indicates that social stigma associated with sex life is common among the participants and if a man does not have the ability to perform sexually as is expected of a man, then he is often considered as a person of bad character.

The most prominent cause of social stigma associated with sex life was found to be the idea that men should dominate women in sexual thing. If the men are not performing adequately during intercourse, it is thought to be shameful and a matter of ridicule. Majority participants reported that if they were ever found out of having these problems, they would be subjected to social and familiar shame and stigma; they identified it to be an important reason of domestic dispute with wife, and that it may lead to wives leaving them. One participant, 27-year-old three-wheeler vehicle driver, said:

One has to be physically strong in order to feel pleasure. If there is strength, everything is fine. If one has no strength, or is sexually weak; one can’t have intercourse for too long. In that case neither husband nor wife gets pleasure. If partners can’t satisfy each other there are fights, arguments, upsets. If people get to know it is embarrassing.

### Availability of SRH service providers and perception of urgency for treatment

The study findings suggest that a range of formal and informal healthcare providers provide SRH services to married men in the three study areas. Informal healthcare providers are those who do not have a formal medical degree but provide treatment mostly in the rural areas and urban slums in Bangladesh [17]. These providers range from village doctors (sometimes called rural medical practitioners or *palli chikitsok*) to faith healers (*hujur, pir and fakir*), drug store salespeople, street-based medicine sellers, *kabiraj* and *hakim* (herbalists). Of these informal providers, drug store salespeople were found to be the most available type of providers (29.7%) followed by village doctors (21.8%) and homeopaths (21.8%) in the study sites (Table 3). In terms of popularity, village doctors were found to be the most popular (28.1%) followed by drug store salespeople (23.6%). It is interesting to note that only 9.2% of the participants reported that a certified doctor (a doctor with the Bachelor of Medicine, Bachelor of Surgery (MBBS) degree; also locally called MBBS doctor) is available in their areas and only 19.4% of the participants reported they are the most popular type of providers (Table 3).

**Table 3:** Available and popular SRH service providers in the study sites

Provider type	Participants reported as available in their village* (*n* = 293)	Participants rated as the most popular* (*n* = 242)
	Frequency (percentage)	Frequency (percentage)
Drug store salespeople	87 (29.7)	57 (23.6)
Village doctor	64 (21.8)	68 (28.1)
Homeopath	64 (21.8)	31 (12.8)
Hujur/pir fakir/shrine	44 (15.0)	2 (0.8)
Kabiraj	36 (12.3)	19 (7.9)
Pharmacy with MBBS doctor	27 (9.2)	47 (19.4)
Govt. health center/hospital	15 (5.1)	11 (4.5)
Hakim	6 (2.0)	3 (1.2)
Street vendor of medicines	6 (2.0)	3 (1.2)
Friends and family	6 (2.0)	0 (0)
Ojha/baidha/totka	4 (1.4)	0 (0)
Private clinic	3 (1.0)	1 (0.4)
NGO clinic	3 (1.0)	0 (0)
Family planning worker	1 (0.3)	0 (0)

*Multiple responses

Qualitative findings suggest that though there are a range of SRH service providers available in the study sites, not all of the participants visited them. If the sexual illnesses were actively hampering sex life by compromising sexual performance of the men, then these were perceived as illnesses which required urgent treatment. In majority of the cases, forty five of the sixty IDI participants reported that, they only started to get treatment for illnesses when their wives found out and persistently pushed them to get treatment. In fifteen cases domestic disputes led to men seeking treatment for their illness to have a better sex life. A participant, 29-year-old fisherman, said:

I have had itching in my penis for a long time, about six years now. It comes and goes. I never felt the need to get any medication for it. I also have another illness; it is troubling me for six months now. I lose semen too quickly during sex. I have sought treatment for this from the local doctor (chamber in local pharmacy). My wife kept telling me to get treated.

The above case study suggests that the participant was suffering from losing semen too quickly during sex for a long time; however, he did not seek treatment for it. He only sought treatment when his wife told him to get treated. Majority of the qualitative participants also opined that the perception of losing control over wife, unhappy married and sex life and fear of rejection and abandonment, made men want to seek treatment.

### Choice of providers and the influencing factors: privacy, popularity and satisfaction with treatment

**Fig. 1:**
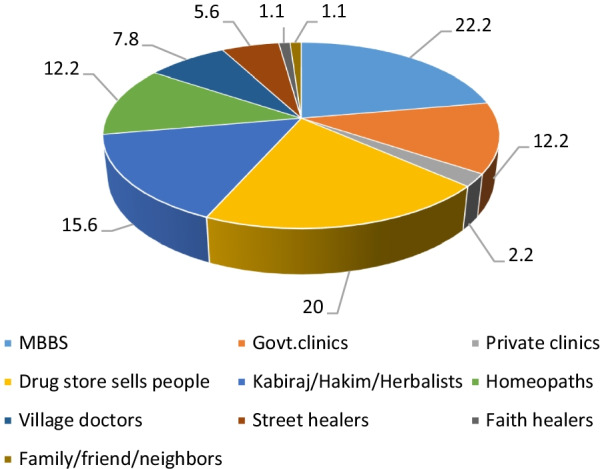
Providers who are the first point of contact for any self-reported SRH concern. The figure displays the percentages of participants visiting a type of health care provider as the first point of contact for any self-reported SRH concern. The percentages were derived from responses (single response question) of 151 survey participants who had suffered from and received treatment for any SRH concern (out of the total 311 ever-married men who participated in the survey). Majority of the participants visited independently practicing MBBS doctors (22.2%), followed by drug store salespeople (20%), followed by traditional kabiraj/hakim/herbalists (15.6%), then homeopaths (12.2%), SRH providers in Govt. clinics (12.2%), village doctors (7.8%), street healers (providers who are canvassers, selling herbal medicines on the streets) (5.6%), doctors in private clinics (2.2%), followed by faith healers (religious healers who claim to heal using holy scriptures) (1.1%), and some (1.1%) participants were found to turn to family, friends and neighbors for advice on home remedies before seeking care from any other formal/informal providers

Over half (59.6%) of the 151 survey participants who suffered from any SRH concern received treatment. For majority (63.4%) of them, the first point of contact was an informal SRH service provider (Fig. 1). Among informal providers, drug store salespeople were mostly visited (20%) followed by traditional herbalists (15.6%), homeopaths (12.2%) and village doctors (7.8%). Those who started their treatment with a formal provider, majority visited independently practicing MBBS doctors (22.2%).

Table 4 shows that participants sought multiple options to treat their concerns and in many cases more than one provider. For example, 51 participants suffered from shortened duration of sexual intercourse (premature ejaculation/ejaculation before coitus), of whom 29 consulted a first provider, of whom 12 also consulted a second provider, and 6 a third provider.

**Table 4:** Treatment pathways: types of providers consulted, and the order in which they were consulted, by the study participants for their leading SRH concerns

Self-reported SRH concerns	Number of men	1st provider	2nd provider	3rd provider
		(*n* = 29 men)	(*n* = 12 men)	(*n* = 6 men)
Shortened duration of sexual intercourse (premature ejaculation/ejaculation before coitus)	51 men reported, of whom 29 consulted providers, of whom 12 also consulted a second provider and 6 a third provider	MBBS Doctor (9) Drug store salespeople (5) Kabiraj/Hakim (4) Village doctor (3) Homeopath (3) Street Healer (3) Faith Healer (2)	MBBS Doctor (5) Homeopath (2) Govt. Hospital (2) Street Healer (2) Village Doctor (1)	Drug store salespeople (2), Kabiraj/Hakim (2) Village Doctor (1) Street Healer (1)
		(*n* = 23 men)	(*n* = 10 men)	(*n* = 5 men)
Loss of semen before or after urination	36 men reported, of whom 23 sought treatment, of whom 10 consulted a second provider and 5 a third provider	Homeopath (5) kabiraj/hakim (5) Drug store salespeople (4) MBBS doctor (4) Others (5)	MBBS doctors (3) Homeopath (1) Kabiraj (1) Hujur (1) Others (4)	Village doctor (2) MBBS doctor (1) Others (2)
		(*n* = 22 men)	(*n* = 8 men)	(*n* = 3 men)
Burning or pain when urinating	35 men reported, of whom 22 sought treatment, of whom 8 visited a second provider and 3 a third provider	Drug store salespeople (5) Govt. Hospital (4) MBBS Doctor (3) Kabiraj/Hakim (2) Homeopath (2) Others (6)	MBBS Doctor (3) Drug store salespeople (2) Street Healer (1) Others (2)	MBBS Doctor (1) Homeopath (1) Homeopath (1)
		(*n* = 13 men)	(*n* = 2 men)	(*n* = 1 men)
Itching or burning in the genital area	19 men reported, of whom 13 sought treatment, of whom 2 visited a second and 1 visited a third provider	MBBS Doctor (6) Drug store salespeople(3) Street Healer (1) Kabiraj/hakim (1) Others (2)	MBBS doctor (1) Drug store salespeople (1)	Kabiraj/Hakim (1)
		(*n* = 3 men)	(*n* = 2 men)	(*n* = 2 men)
Worries about penis size	8 men reported, of whom 3 received treatment, of whom 2 visited a second and third provider	Kabiraj/Hakim (1) Drug store salespeople (1) Street Healer (1)	Govt. Health Center (1) Private Clinic (1)	Drug store salespeople (1) Govt. Health Center (1)

Qualitative findings suggest that many of the participants tried home remedies such as eating eggs for energy, drinking psyllium husk (Ishabgul) with water for energy, having Neem leaves soaked water for treating infections, and, drinking more water to reduce burning sensation during urination, to treat their SRH conditions before visiting any healthcare provider. In all the cases, when it did not work, they sought treatment from healthcare providers over time. Primarily, most of them visited village doctors and allopathic drug store salespeople. These informal providers were chosen because they were well known in the area, the cost of treatment was low and they were easily accessible to the participants compared to formal healthcare providers. Many of the times, these informal providers were recommended by a peer or friend with similar illness who had visited the provider; this also assured the participants that the visit would be discreet and the doctor would not judge. One participant, 27 years old, said:

I am suffering from burning during urination for almost 2 years now. I took glucose and saline for this problem. When I was really suffering badly 3 months ago, I went to my village doctor ‘XXX’ to get treatment. The doctor was an experienced allopathic doctor. He gave very good treatment for my problem.

The above quote indicates that the participant tried home remedies first for his sufferings associated with burning during urination. However, when the home remedies did not work to improve the condition, he visited the village doctor and got treatment.

Local kabiraj and homeopathic doctor were also found to be very popular among the participants, opted by twenty-two IDI participants among the sixty. They were available, lived in the same community, known by name to many and easily accessible; they spoke in the same cultural language when explaining the meaning and causes of the illness and exuded expressions of non-judgment and understanding of the illness from the patient perspectives, making them very trustworthy to all care seekers. Participants mentioned taking treatment from them frequently for illnesses that resulted in weakness and poor sexual performance, such as shortened duration of intercourse, loss of semen before and after urination resulting in physical weakness, thin/poor amount of discharged semen, premature ejaculation, inability to get an erection and nocturnal emissions. However, analyses of the responses reveal that the illnesses were not cured by such treatments of the informal providers; rather the symptoms dissipated with a consequent relapse in the future. One participant, 35-year-old farmer, said:

Homeopathic and herbal medicines that I took worked for some time. I bought and took those for a while. However, when I did not get well, I stopped taking those medicines.

Many of the participants reported having persisting symptoms which made them change health care provider multiple times; for example–trying out new herbal medicines from different kabirajs, switching to allopathic medicines bought from pharmacy, and when none of these worked, seeking health care from local doctors who reportedly had medical degree or local hospitals. One participant, 35 years old, said:

I suffer from loss of semen before and after urination. I took treatment from Ayurvedic, Allopathic & Homoeopathic doctors for this illness. An Ayurved (Kobiraj) gave me medicines like halua (Paste type medicine made of herbal and medicinal plants), herbal pill and some medicines from Hamdard (An ayurved pharmaceutical company). When these didn’t work I went to the Allopathic doctor. He first gave me antibiotic tablets (I don’t remember the name), along with a multivitamin named Best of Gold. Even these didn’t work. Then he gave me 3 injections, followed by another 7 injections but there was no result. Then I went to the Homoeopathic doctor who gave me some pills & some liquid medicine but got no improvement. Finally I went to Shattola Hospital.

The above case study suggests that the participant went to several informal providers and took medicines according to their advice. However, when the condition did not improve, he finally visited the hospital. This same treatment pathway was adopted by many of the participants.

Only a few of the IDI participants, eleven out of the sixty went to formal providers, who were doctors with medical degree (MBBS degree) for treatment. They mostly visited private clinics, only three visited public hospitals in the area. Participants, who had long-standing illness and were not satisfied with the explanation of causality and treatment from informal providers, paid money for diagnostic tests and visited formal providers for treatment. The participants, who did visit a formal provider, reported they were satisfied with the treatment and advice, as they helped curing/alleviating symptoms. One participant, 39 years old, said:

I used to suffer from loss of semen during urination. I went to Dr. Bashir (pseudonym) for treatment. He gave me E-Cap & some other tablets. The medicines worked. He prescribed me medicines after figuring out my illness. He is a good, well-mannered doctor. I went to him thrice & took medicines properly. Now I am cured.

## Discussion

Our study findings show that in Bangladesh men’s various sexual and reproductive health concerns are embedded in psychosocial and cultural concerns about their masculinity and expectations of themselves as sexual beings, with worries about performance, loss of semen and virility being dominant concerns. Sexually transmitted infections (STIs) were also mentioned as a concern, but ranked lower. Men’s health-seeking behaviors related to SRH are shaped by individual, provider and broader contextual factors. Their knowledge and ideas about STDs/STIs and psychosexual dysfunctions affect perceptions about sound SRH and health-seeking behavior whereas availability, accessibility, acceptability and quality of health care providers play important role in SRH service utilization. These individual and provider related factors are influenced by the broader sociocultural norms, environmental factors and government policy and provisions for health care. Amaro and Gornemann [25], in their work, have shown that health care utilization for STDs is influenced by patient characteristics (socio-demographics, knowledge and skills, beliefs attitudes and values, psychological and psychosocial factors) and provider characteristics (availability, accessibility, quality of service, knowledge and skills of provider). Therefore, the complexity of tackling men’s SRH problem in Bangladesh highlights the need of adopting interventions at three different levels—(1) at the patient level, (2) at the provider level and (3) at the national level. In the Indian subcontinent including Bangladesh, there has been a general lack of SRH interventions targeting men [10, 1]. Sometimes quite simple interventions such as conveying SRH information or an opportunity to talk to a trained health care providers can resolve problems that can be a source of considerable distress to men and their partners [27]. It is therefore important that men receive appropriate information and education on SRH and related conditions in order to raise their awareness and to improve care seeking in SRH illnesses. Health care providers including informal providers need to be skilled and sensitized, so they can provide quality services and convey correct messages on SRH to men. National policies and provisions of health services need to devise strategy to manage and improve access to facility level health care.

The participants had lack of knowledge and varied misconceptions about the conditions that affect their SRH. Particularly, when it came to psychosexual dysfunctions, there was a lack of recognition that it was involved with the psychology of the individual not only physical. This lack of knowledge may be attributed to lower or no education of the study participants. Among all the survey participants, 34.1% did not have any formal education whereas 19.6% did not complete primary level education. Besides, most of the sample participants belonged to poor socioeconomic class and live in rural areas or informal settlements, where electronic devices such as television are not available in every household and less information is dispersed through digital and print media. Similar findings were reported from surveys in India, which revealed that men with higher levels of education and economic status, and those living in urban areas, have better knowledge of reproductive health matters, seek treatment more frequently and are more likely to protect themselves against STDs than other men [28].

Another important factor that influences men’s psychosexual concerns and SRH related treatment seeking is the ideals of hegemonic masculinity. Hegemonic masculinity was understood as ‘the pattern of practice that allowed men’s dominance over women to continue’ [29]. The social-construct of ‘being a man’ dictates need for men to dominate women during sexual intercourse and assume the dominant role in the family. This is consistent with the findings of this study, where participants were found more concerned when they suffered from sexual/psychosexual dysfunctions that hampered their conjugal life; with regular domestic altercations with wife, unhappy conjugal relationship or wife threatening to leave, the major concern for men was the repercussion from wife, family and society tarnishing his claims of ‘masculinity.’ These ideas drove men to prioritize treatment, rather than the actual understanding of the clinical illness. This may also be the reason why informal providers, who utilize tactics and messages such as medicines to improve sexual prowess or portray STDs as problems related to sexual intercourse and semen loss that can be cured by their medicines, attract more patients than formal providers who solely focus on clinical treatment. On the other hand, these ideas drive men to lash out and resort to domestic abuse to hide their vulnerability through display of power, as found in the study; similarly, Hawkes & Collumbien and Silverman et al. [10, 20] also report that men who suffer from SRH problems but do not disclose to wives are more prone to physically abuse their partners compared to men who disclose.

We also found that the main existing sources of information for STDs and other psychosexual dysfunctions in the locality were the local drug store salespeople and traditional healers instead of professional health care providers. The informal providers were approached more compared to formal providers because they were available and financially more accessible than formal providers. Ahmed et al. [30] report that, in Bangladesh, there are approximately 5 qualified physicians per 10,000 population, with gross imbalance in distribution favoring urban areas, whereas there are around 12 unqualified village doctors and 11 unqualified drug store salespeople per 10,000 population, and on top of that there are abundant street-sellers of medicine, herbalists and faith healers. Given this shortage of formal providers and mushrooming of informal providers, it is quite realistic that many men will solve their SRH concerns by consulting with the informal providers. A study conducted by Ahmed et al. on care-seeking behaviors for STDs among male clients of female sex workers in Bangladesh found that 52.3% of the participants had received treatment from drug store salespeople and local healers, whereas only 37.4% received treatment from qualified medical professionals [17]. Similar reports of men underutilizing formal health care services due to limited financial resources were found in other studies [14–16, 18, 31, 32]. Inaccessibility to good quality formal health care due to unavailability of formal health care providers and financial constraints has profound implications for equity in health care and attainment of the SDGs by the country. Wagstaff [33], lead economist for World Health Organization (WHO), reported that, in the developing countries, high income was associated with frequent use of formal private and public sector health care, whereas low income, which was characteristic of the study population of our study who were majorly daily wage earners, was associated with frequent use of informal/traditional health care.

Men’s beliefs, attitudes and health-seeking behavior have dire implications on the health and sexual and reproductive rights of women [7]. Men without knowledge of symptoms or under misconceptions will continue to delay seeking help and propagate infection to the partner; they often fail to recognize psychosexual dysfunction as a clinical problem and delay/avoid seeking appropriate treatment [17, 28, 34]. Men who resort to violence negatively affect health-seeking behavior and reproductive rights of women. Therefore, to preserve women’s SRHR as well as gender equity in health, it is very important to focus on and include men in interventions. The 1994 ICPD had highly emphasized on interventions involving men, to achieve gender equality [35]. Since then, many programs across the globe have had positive results which include ‘Men Make a Difference’ World AIDS Campaign in 2000–2001; ‘White Ribbon Campaign’ which is a male movement against violence against women and girls (VAWG) in 30 countries; and ‘Men as Partners’ in South Africa which promotes gender awareness and have evolved to try an effect changes in broader gender norms and national policy [35, 36]. Gender transformative messaging in these programs was effective in promoting gender equality and better health outcomes, and program messages tailored to address men’s values and needs, in accordance with their sociocultural contexts were more successful in reaching men positively [35, 37–40].

Several limitations of the study should be noted. Firstly, we recruited ever-married men aged 15–49 years only from 3 districts of Bangladesh. Thus, the study findings cannot be generalized for all married men of the country. Due to the sensitivity of the topic, many men might not have shared their experiences and sufferings of SRH with the data collectors. Another limitation we must acknowledge is that the data used in this paper are old as this research was conducted in 2008–2009. However, given the lack of research and knowledge gap on men’s SRH in Bangladesh, this paper contributes to the scientific studies by providing evidence on men’s psychosexual concerns, their worries about sexual dysfunction and abilities, well-being and choice of providers. We strongly feel that the findings presented in this paper would help generate appropriate policies and interventions to improve SRH status of men–women alike in Bangladesh.

## Conclusion

This study shows that married men in the study sites suffer a range of SRH conditions and psychosexual anxieties. The existing health care system does not adequately address their SRH needs and miss out the mental, emotional and social dimensions of SRH and well-being of men which are very real. It is critical that the public health programs and services in Bangladesh accommodate the SRH needs of men by providing improved SRH services including psychosexual counseling and counseling for STIs at the facility level. National policies and provisions of health services need to devise strategy to manage and improve practices of informal providers as well as address issues like shortage of qualified health professionals and high cost of facility level health care.

## Data Availability

The datasets used and/or analyzed during the current study are available from the corresponding author on reasonable request.

## Abbreviations

AIDS: Acquired Immunodeficiency Syndrome
BRAC JPGSPH: BRAC James P Grant School of Public Health
HIV: Human Immunodeficiency Virus
HIV/AIDS: Human Immunodeficiency Virus /Acquired Immunodeficiency Syndrome
HPV: Human Papilloma Virus
ICPD: International Conference on Population and Development
IDIs: In-depth Interviews
MBBS: Bachelor of Medicine, Bachelor of Surgery
PAHO: Pan American Health Organization
SDGs: Sustainable Development Goals
SPSS: Statistical Package for the Social Sciences
SRH: Sexual and Reproductive Health
SRHR: Sexual and Reproductive Health and Rights
STDs: Sexually Transmitted Diseases
STIs: Sexually Transmitted Infections
STI/STDs: Sexually Transmitted Infections/Diseases
UHC: Universal Health Coverage
UTIs: Urinary Tract Infections
VAW: Violence against Women
VAWG: Violence against Women and Girls
WAS: World Association for Sexology
WHO: World Health Organization
